# Dietary fat intakes, lipid profiles, adiposity, inflammation, and glucose in women and men in the Framingham Offspring Cohort

**DOI:** 10.3389/fphys.2023.1144200

**Published:** 2023-05-03

**Authors:** Ioanna Yiannakou, Mengjie Yuan, Xinyi Zhou, Martha R. Singer, Lynn L. Moore

**Affiliations:** ^1^ Preventive Medicine and Epidemiology, Department of Medicine, Boston University Chobanian and Avedisian School of Medicine, Boston, MA, United States; ^2^ Doctoral Program in Biomedical Sciences, Nutrition and Metabolism, Boston University Chobanian and Avedisian School of Medicine, Boston, MA, United States

**Keywords:** saturated fat, unsaturated fat, adiposity, inflammation, lipids, lipid particles, sex, cohort study

## Abstract

**Introduction:** The role of dietary fat in the evolution of cardiometabolic disorders is highly controversial. As both dietary intake and the development of cardiometabolic risk differ by sex, we evaluated sex-specific differences in the associations between dietary fats (saturated and unsaturated) and four key cardiometabolic risk factors—lipid profiles, body fat, inflammation, and glucose regulation.

**Methods:** We included 2391 women and men aged ≥30 years in the prospective Framingham Offspring Cohort. Weight-adjusted dietary fats (saturated, monounsaturated, and polyunsaturated fats, including omega-3 and omega-6) were derived from 3-day dietary records. Analysis of covariance was used to derive adjusted mean levels of all outcomes.

**Results:** In both men and women, intakes of saturated and monounsaturated fats were inversely associated with TG:HDL ratio (*p* < 0.02 for both types of fat). In women, higher omega-3 and omega-6 PUFAs were also inversely associated with TG:HDL (*p* < 0.05 for both), but for men, only omega-3 PUFAs were associated (*p* = 0.026). All types of dietary fat were beneficially associated with larger HDL particle sizes in both men and women, while only saturated and monounsaturated fats were associated with larger LDL particles in men. In addition, saturated and monounsaturated fats were associated with statistically significantly higher concentrations of HDL and lower concentrations of LDL and VLDL particles in both sexes, while polyunsaturated fat had favorable associations in women only. Saturated fat also had beneficial associations with three measures of body fat. For example, women with the highest (vs. lowest) saturated fat intake had a lower BMI (27.7 ± 0.25 vs. 26.2 ± 0.36 kg/m^2^, *p* = 0.001); findings were similar in men (28.2 ± 0.25 vs. 27.1 ± 0.20, *p* = 0.002). Unsaturated fats had beneficial associations with body fat primarily in women. Finally, omega-3 PUFAs among women were inversely associated with interleukin-6 levels. There was no association between dietary fat intake and fasting glucose levels in either women or men.

**Discussion:** In sum, we found no evidence of an adverse association between dietary fats and several surrogate markers of cardiometabolic health. This study suggests that different dietary fats may have divergent associations with cardiometabolic risk in women and men, perhaps owing to differences in food sources of the same dietary fats.

## Introduction

For decades, the *Dietary Guidelines for Americans* have recommended restricting intakes of total and saturated fats and replacing saturated with unsaturated fats to prevent cardiovascular disease (CVD). The basis for these recommendations is the long-standing “diet-heart” hypothesis which proposed that higher saturated fat intakes would increase serum low-density lipoprotein cholesterol (LDL-C) levels, thereby increasing CVD risk (Forouhi et al., 2018). In recent years, more attention has been focused on the association between dietary fats and several surrogate markers of CVD, including lipid ratios, lipid particles, and other cardiometabolic risk factors such as inflammation and adiposity.

Saturated fat intakes have been shown to be linked with higher LDL-C levels but also with higher high density lipoprotein cholesterol (HDL-C) levels and, by some, with lower triglyceride (TG) levels (Mensink et al., 2003), although evidence on the latter is inconsistent (DiNicolantonio and O’Keefe, 2018). The ratio of triglycerides-to-high density lipoprotein-cholesterol (TG:HDL) levels has been shown to more strongly predict CVD risk than individual lipids (Chen et al., 2022). In addition, a preponderance of small, dense lipid particles has been shown to be more pro-atherogenic than larger buoyant particles (Krauss, 2022), and in some studies, higher saturated fat intakes have been associated with larger LDL particle sizes (DiNicolantonio and O’Keefe, 2018). The particular food sources of saturated fat may also have important effects on particle sizes and concentrations (Yuan et al., 2022). Lastly, the interaction of other macronutrients, especially carbohydrates, with lipid metabolism may also play a key role in the effects of dietary fat intake on dyslipidemia (Parhofer, 2015).

Evidence suggests that different types of dietary fats have divergent associations with adiposity, inflammation, and glucose, key markers for the onset of several chronic diseases. While animal studies have shown that higher intakes of polyunsaturated fat have anti-inflammatory (Fritsche, 2015) and anti-obesity effects (Buckley and Howe, 2009), the findings in humans are weaker. More research on the associations of polyunsaturated fats, including omega-3s and omega-6s, on cardiometabolic risk, is needed. Saturated fat is usually associated with the consumption of more energy-dense foods. However, a recent systematic review of a few trials found that a reduction in saturated fat intake vs. a usual diet led to small reductions in body mass index (BMI) (Hooper et al., 2020). Evidence from long-term studies is lacking.

Given that the lipoprotein metabolism and body fat distribution differ between women and men (Knopp et al., 2005; Varlamov et al., 2015), sex-specific differences in the associations of dietary fats with lipoproteins and adiposity would not be surprising. This study evaluated sex-specific associations between dietary intakes of saturated, monounsaturated, and polyunsaturated (including total, omega-3, and omega-6) fats and cardiometabolic risk factors in the Framingham Offspring Cohort. Specifically, we separately examined associations between different types of dietary fats and lipid levels, particle concentrations and sizes, body fat, biomarkers of inflammation, and fasting glucose in women and men.

## Materials and methods

### Study population

**FIGURE 1:**
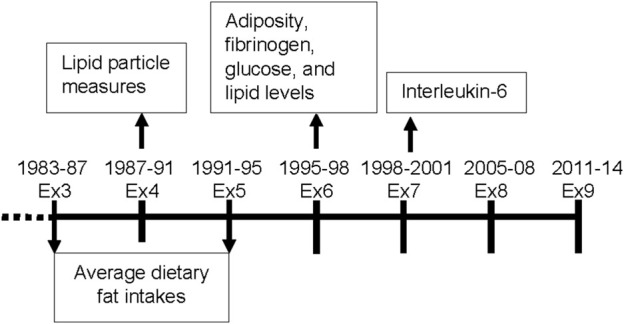
Timeframe for data collection of dietary variables and cardiometabolic outcomes for these analyses in the Offspring Cohort. Ex = exam.

The Framingham Offspring Cohort enrolled 5124 adults in 1971 who were the offspring of participants in the original Framingham Heart Study. Approximately every 4 years, participants were asked to complete questionnaires on health status, lifestyle, and demographic information and to undergo anthropometric measurements and blood tests. Dietary information from food records was collected and averaged from exams three (1983-87) to five (1991-95), so we considered exam five to be the baseline for these analyses. Figure 1 shows the timeframe of data collection for the exposure and outcomes for these analyses. The Boston University/Boston Medical Center Institutional Review Board approved the study protocols, data collection, and data analysis. An earlier published preprint includes some of the results in this manuscript (Yiannakou et al., 2022). All participants provided written informed consent.

**FIGURE 2:**
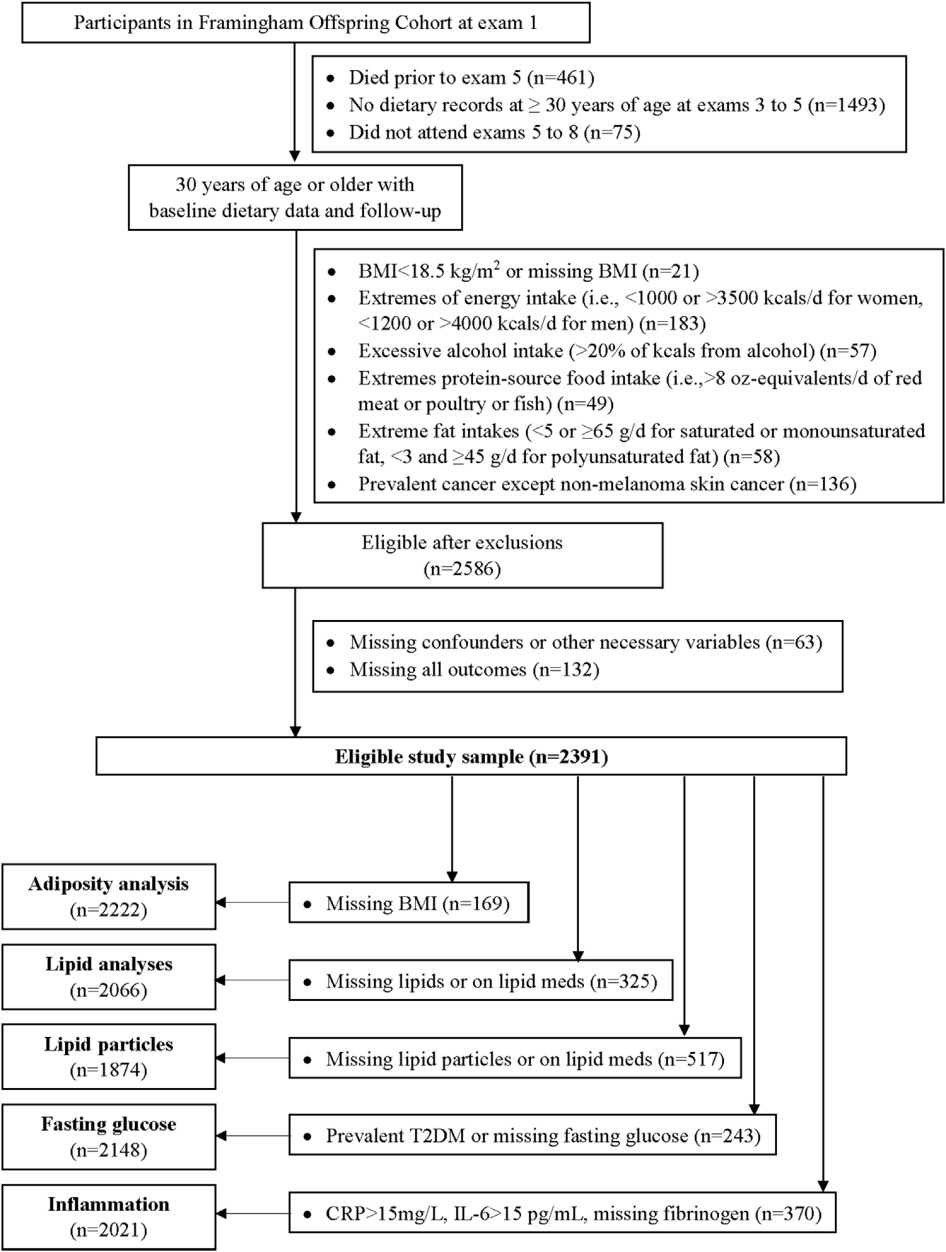
Flow diagram of the analyses in the Offspring Cohort. BMI = body mass index, T2DM = type 2 diabetes mellitus, CRP = c-reactive protein, IL-6 = interleukin-6.

Figure 2 provides inclusion and exclusion details for the current analyses. Of the 5124 participants at the first examination visit, we included 3095 who survived up to exam 5, provided at least one set of 3-day dietary records at ≥30 years of age between exams 3 and 5, and attended follow-up exams. Examination visit 5 served as the baseline visit for these analyses. We excluded subjects at baseline with missing BMI or a BMI <18·5 kg/m^2^, prevalent cancers, alcohol consumption >20% kilocalories daily, or extreme intakes of total energy (<1,000 or >3500 kcal/d for women, <1,200 or >4,000 kcal/d for men), dietary fats, or red meat, poultry, or fish. This left 2586 participants. We further excluded 63 who were missing potential confounding variables and 132 who were missing all of the outcome measures of interest (i.e., body fat, lipids, lipid particles, glucose, and inflammatory biomarkers), leaving a maximum of 2391 participants for these analyses. Sample sizes for individual cardiometabolic risk factors differ and are shown in Figure 2.

### Dietary assessment

Approximately 16,000 days of diet records were collected, with each set including two weekdays and one weekend day. Instructions were provided by a trained nutritionist and included the use of two-dimensional food models to estimate portion sizes. Nutrient composition of the diet was derived by entering the diet records into the Nutrition Data System (NDS) of the University of Minnesota, version 23 (Schakel et al., 1988). Intakes in each USDA food group were derived by linking food code data from the NDS with USDA food codes using the MyPyramid Equivalents Database, version 06A (U.S. Department of Agriculture and U.S. Department of Health and Human Services, 2020). Mean intakes of foods and nutrients were estimated from all days of diet records. Exposure variables for these analyses included saturated, monounsaturated, and polyunsaturated fat, including omega-3 and omega-6 PUFAs. The omega-3 PUFAs available through the NDS system are 18:3 (linolenic acid), 18:4 (stearidonic acid), 20:5 (eicosapentaenoic acid), 22:5 (docosapentaenoic acid), and 22:6 (docosahexaenoic acid). The available omega-6 PUFAs include 18:2 (linoleic acid) and 20:4 (arachidonic acid).

### Outcomes

Routine laboratory assessments for lipids at exam six were conducted following a 8-h fast. Plasma lipid levels (LDL-C, HDL-C, TG) were determined in the Framingham lipoprotein cholesterol laboratory following Centers for Disease Control guidelines (McNamara and Schaefer, 1987) for measured or estimated cholesterol content. Lipoprotein particle sizes and concentrations for HDL, LDL, and very low density lipoprotein (VLDL) were measured by nuclear magnetic resonance spectroscopic assay. The average weighted lipoprotein particle size (nm diameter) was computed as the sum of the size of each subclass multiplied by the percent of its relative mass as estimated by the amplitude of its nuclear magnetic resonance signal. Particle concentrations are expressed as mmol/L. Since lipid particles were only measured at exam four, and thus they were analyzed cross-sectionally here (Figure 1).

Adiposity, fibrinogen levels and 8-h fasting glucose were assessed at exam 6, 4 years after baseline. The first available measure for Interleukin-6 (IL-6) after baseline was at exam 7 and used in this analyses (Figure 1). General adiposity was measured using BMI and percent body fat. Mean height (from all measures prior to age 60) was used together with weight from exam six to calculate BMI (kg/m^2^). Percent body fat was estimated using a single bioelectrical impedance analysis (BIA-101, RJL Systems) following a previously described validated protocol (Lukaski et al., 1986). Abdominal adiposity was measured using the waist-to-height ratio (WHtR). Waist circumference was measured at the level of the umbilicus during mid-respiration to the nearest 0.25 inch with a cloth tape. Waist circumference was divided by averaged height to calculate WHtR (with missing values at exam 6 being substituted using the mean values from exams 5 and 7). IL-6 and fibrinogen were measured with commercially available enzyme-linked immunoassay kits.

### Potential confounding

We explored several potential confounding factors in the multivariable models. Education level and physical activity were self-reported. Information on education was assessed at exam 2 and classified as high school or above vs. less than high school. Physical activity as assessed by a questionnaire at examination visit 5 (Kannel and Sorlie, 1979) was used to calculate a baseline physical activity index by multiplying daily hours of moderate and vigorous activity by an appropriate weight based on oxygen consumption required for that level of exercise (Kannel et al., 1986). Cigarette smoking status and amount smoked were assessed at every exam by interview, and total pack-years of cigarette smoking were updated at each exam; we used updated data from the baseline exam visit (exam 5) in these analyses. Age at menopause was assessed by interview at each exam (exams 1-9) until the occurrence of menopause. The following dietary factors during exams 3-5 were also explored as potential confounding variables: (a) energy-adjusted (and weight-adjusted) intakes of monounsaturated fat (for total, omega-3, and omega-6 PUFA models), polyunsaturated fat (for SFA and MUFA models), protein, and dietary fiber; (b) servings per day of foods such as fruits, vegetables, and dairy products, (c) energy intake (kilocalories per day); and (d) 2015 Healthy Eating Index (HEI) scores (Krebs-Smith et al., 2018). Given the strong interplay between fatty acid and carbohydrate metabolism, we also explored effect modification and confounding by energy-adjusted and body weight-adjusted carbohydrate intakes. Finally, we explored co-morbidities as of the baseline visit at exam 5 for these analyses, including prevalence of hypertension and diabetes status, as determined at each examination visit, as well as baseline lipid-lowering and antihypertensive medications use.

### Statistical analysis

The intake of each type of dietary fat (grams per day) was adjusted for the participant’s body weight at baseline (exam 5) by adding the residuals from linear regression models to the overall median intake values. The weight-adjusted intakes were compared with energy-adjusted intakes among a subset of participants who were determined to have plausible energy intakes (within 20% of the estimated total energy expenditure (Gerrior et al., 2006)). There were strong correlations between energy-adjusted vs. body weight-adjusted intakes among those with plausible intakes but weaker correlations among those with implausible energy intakes. Thus, we chose to adjust dietary fat intake for body weight rather than energy intake to minimize the impact of biased reporting of energy intake.

We used sensitivity analyses and power considerations to classify each subject’s intake of weight-adjusted dietary fats. Categories of saturated fat intake were as follows: <20, 20–<30, and ≥30 g/day. Monounsaturated fat intakes were classified as <25, 25–<35, and ≥35 g/day, while total polyunsaturated fat intakes were classified as <12, 12–<20, and ≥20 g/day. Intake of omega-3 PUFA was classified as <1, 1–<2, and ≥2 g/day, while omega-6, the predominant PUFA, was categorized as <10, 10–<15, and ≥15 g/day.

We used analysis of covariance (ANCOVA) modeling to estimate adjusted mean lipid levels (HDL-C, LDL-C, TG, and the TG:HDL ratio), lipoprotein particle sizes and concentrations (HDL, LDL, and VLDL), adiposity (BMI, % body fat, waist-to-height ratio), inflammatory markers (IL-6 and fibrinogen), and fasting glucose. Non-normally distributed variables were log-transformed, including TG, IL-6, fibrinogen, and VLDL particle concentrations.

Confounding was assessed by adding each factor one at a time to the age- and sex-adjusted models, then building the model forward by adding each individual confounder singly to the model and avoiding collinearity. Sex-specific final models included age, weight-adjusted carbohydrate intakes, HEI 2015 scores, use of lipid-lowering medications, pack-years of cigarette smoking, baseline BMI, and prevalent diabetes. None of the other potential confounders altered the effect estimates and were thus dropped from the final models. Statistical Analysis Systems software, version 9·4 (SAS Institute, Cary, NC), was used to perform all analyses.

## Results

### Baseline characteristics of participants according to intakes of different types of fats

Sex-specific characteristics according to categories of saturated fat intake are shown in Table 1. Women and men with higher (vs. lower) saturated fat intakes were somewhat younger and had lower BMI and HEI scores at baseline. They also had higher intakes of dairy, nuts and seeds, but not higher intakes of fruits and vegetables, poultry, or fish. Red meat intake was positively associated with saturated fat intake in both women and men. Further, women (but not men) in the highest intake category for saturated fat were slightly more likely to have a higher education level. Men with higher saturated fat intakes were also much more likely to be current smokers (17.1% of men with the highest saturated fat intake vs. 9.9% of those with the lowest intakes)

**TABLE 1:** Sex-specific characteristics of participants according to weight-adjusted intakes of saturated fats.

Participant characteristics	Women			Men		
	Saturated fat intake (g/day)			Saturated fat intake (g/day)		
	<20 (n = 511)	20-<30 (n = 552)	≥30 (n = 237)	<20 (n = 273)	20-<30 (n = 396)	≥30 (n = 422)
	Mean (SE)	Mean (SE)	Mean (SE)	Mean (SE)	Mean (SE)	Mean (SE)
Age	57.6 (0.4)	54.8 (0.4)	52.5 (0.6)	58.7 (0.6)	57.0 (0.5)	53.7 (0.5)
BMI (kg/m^2^)	27.7 (0.2)	25.4 (0.2)	25.4 (0.3)	29.0 (0.2)	27.8 (0.2)	27.3 (0.2)
Smoking (packyears)	20.4 (1.2)	22.2 (1.1)	25.3 (1.7)	27.2 (1.6)	28.4 (1.4)	31.2 (1.3)
Physical activity index (MET-eq/day)	14.4 (0.3)	14.6 (0.3)	13.3 (0.5)	14.8 (0.5)	15.4 (0.4)	15.1 (0.4)
Alcohol, g/day (current drinkers)	8.8 (0.6)	10.1 (0.5)	9.6 (0.8)	16.7 (1.2)	18.0 (1.0)	18.5 (1.0)
HEI-2015 score	63.4 (0.5)	56.0 (0.5)	51.6 (0.7)	61.8 (0.6)	55.7 (0.5)	49.7 (0.5)
Total fat (g/day, weight-adjusted)	73.8 (1.13)	74.9 (1.1)	74.3 (1.7)	74.9 (1.5)	74.2 (1.3)	74.9 (1.2)
Saturated	15.5 (0.16)	24.5 (0.15)	36.6 (0.24)	15.4 (0.31)	25.0 (0.25)	38.8 (0.25)
Monounsaturated	18.3 (0.23)	26.7 (0.22)	36.8 (0.34)	19.2 (0.41)	28.8 (0.33)	39.6 (0.33)
Polyunsaturated fats	11.7 (0.21)	14.8 (0.20)	19.0 (0.31)	12.5 (0.36)	16.1 (0.30)	19.5 (0.29)
Omega-3	1.2 (0.02)	1.5 (0.02)	1.9 (0.03)	1.3 (0.04)	1.5 (0.03)	2.0 (0.03)
Omega-6	10.4 (0.20)	13.2 (0.19)	17.0 (0.29)	11.1 (0.34)	14.4 (0.28)	17.4 (0.27)
Food intakes						
Fruit/Vegetables (cup-eq/day)	3.1 (0.06)	2.9 (0.06)	3.0 (0.09)	3.4 (0.10)	3.3 (0.08)	3.2 (0.08)
Meat, poultry, fish (oz-eq/day)	4.2 (0.08)	4.3 (0.08)	5.0 (0.11)	5.6 (0.13)	5.7 (0.11)	6.4 (0.11)
Red meats (oz-eq/day)	1.3 (0.05)	1.9 (0.05)	2.5 (0.08)	2.0 (0.10)	2.8 (0.08)	3.5 (0.08)
Poultry (oz-eq/day)	1.7 (0.05)	1.3 (0.05)	1.2 (0.08)	2.0 (0.09)	1.7 (0.07)	1.6 (0.07)
Fish (oz-eq/day)	1.2 (0.05)	1.1 (0.05)	1.2 (0.07)	1.7 (0.08)	1.3 (0.07)	1.3 (0.07)
High omega-3 fish (oz-eq/day)	0.3 (0.02)	0.2 (0.02)	0.2 (0.03)	0.3 (0.03)	0.2 (0.03)	0.2 (0.03)
Nuts and seeds (oz-eq/day)	0.2 (0.03)	0.4 (0.03)	0.7 (0.04)	0.4 (0.05)	0.6 (0.04)	0.7 (0.03)
Dairy (cup-eq/day)	1.0 (0.03)	1.3 (0.03)	1.6 (0.05)	1.0 (0.05)	1.4 (0.05)	1.9 (0.04)
Education level (column %, >high school)	57.3	61.6	63.7	67.8	61.6	67.5
Current smokers (column %)	14.7	17.0	18.6	9.9	14.4	17.1

Mean fat intakes (exams 3 and 5) were adjusted for weight using residuals from a linear regression model. Analyses were adjusted for age, except analyses for age. BMI, body mass index; Mets, metabolic; eq, equivalents; HEI, healthy eating index; and oz, ounces.

Sex-specific baseline characteristics associated with categories of monounsaturated fat intake are shown in Supplementary Table S1. Results for both women and men are very similar to those for saturated fat intakes. Supplementary Table S2 shows the participant characteristics associated with intakes of total polyunsaturated fat. Women, in particular, with higher intakes of polyunsaturated fat, had higher education levels. Both women and men with higher intakes of polyunsaturated fat had slightly higher mean diet quality scores on the 2015 HEI, higher intakes of fruits and vegetables, and higher intakes of all protein food sources. Further, women and men with higher polyunsaturated fat consumption were substantially less likely to be current smokers (i.e., 10.8% vs. 18.5% for women; 10.6% vs. 19.2% for men).

### Dietary fat intakes and lipid profiles

**FIGURE 3:**
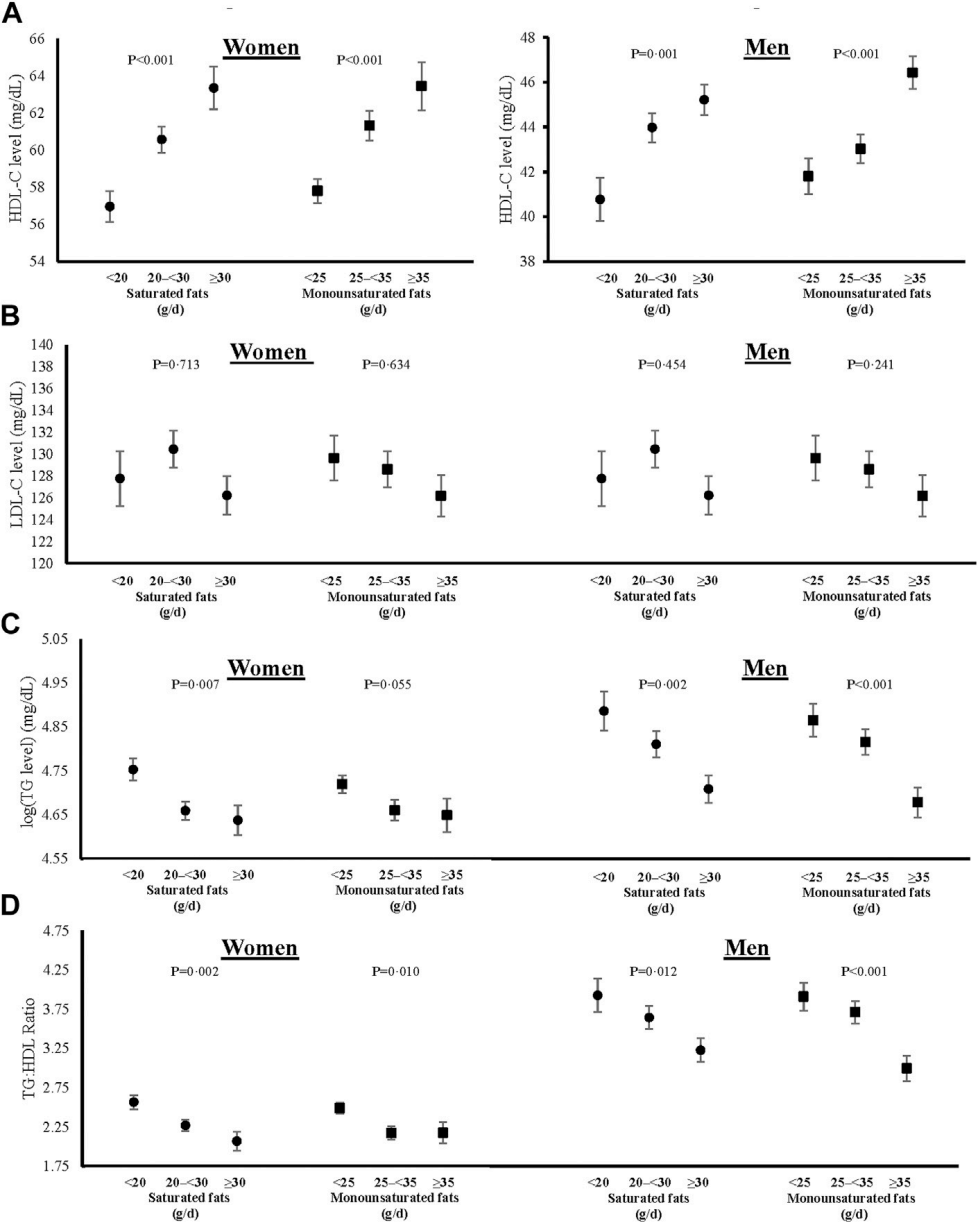
Associations of saturated and monounsaturated fat intakes with blood levels of HDL-C **(A)**, fasting LDL-C **(B)**, log-transformed fasting TGs **(C)**, and TG:HDL ratio **(D)** in women and men. All models were adjusted for age, weight-adjusted carbohydrate intakes, HEI 2015 scores, use of lipid-lowering medications, pack years of cigarette smoking, baseline BMI, and prevalent diabetes. HDL-C = high density lipoprotein cholesterol, LDL-C = low density lipoprotein cholesterol, log (TG) = logarithmic transformed triglycerides, TG:HDL = triglyceride to high density lipoprotein ratio, HEI = Healthy Eating Index, and BMI = body mass index.

**FIGURE 4:**
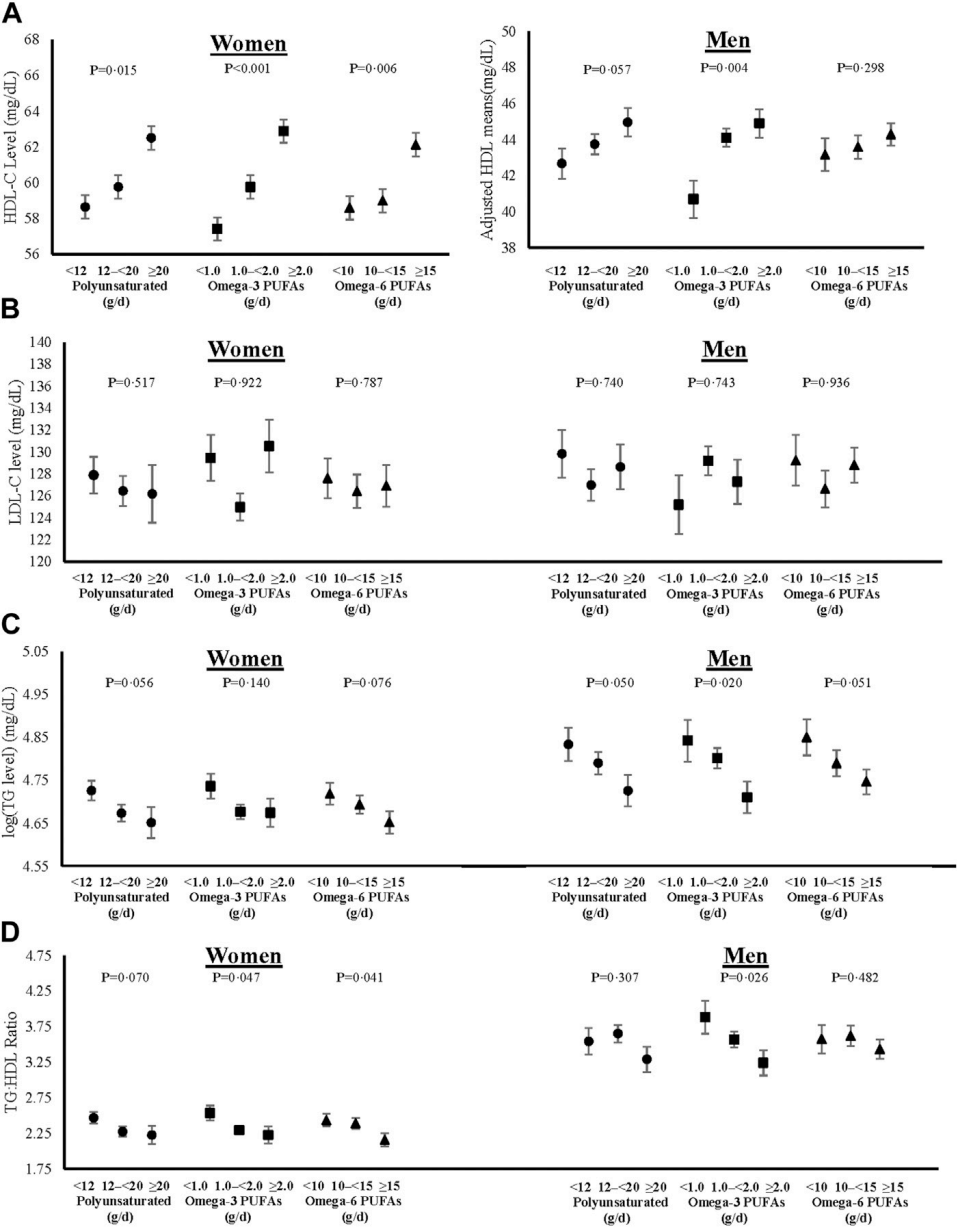
Associations of total, omega-3 and omega-6 polyunsaturated fat intakes on blood levels of HDL-C **(A)**, fasting LDL-C **(B)**, log-transformed fasting TGs **(C)**, and TG:HDL ratio **(D)** in women and men. All models were adjusted for age, weight-adjusted carbohydrate intakes, HEI 2015 scores, use of lipid-lowering medications, pack years of cigarette smoking, baseline BMI, and prevalent diabetes. HDL-C = high density lipoprotein cholesterol, LDL-C = low density lipoprotein cholesterol, log (TG) = logarithmic transformed triglycerides, TG:HDL = triglyceride to high density lipoprotein ratio, PUFAs = polyunsaturated fatty acids, HEI = Healthy Eating Index, and BMI = body mass index.

Sex-specific adjusted mean levels of plasma lipids associated with intake categories of saturated and monounsaturated fats are shown in Figure 3, and those for polyunsaturated fats (including omega-3 and omega-6 PUFAs) in Figure 4. The final models were adjusted for age, weight-adjusted carbohydrate intakes, HEI 2015 scores, use of lipid-lowering medications, pack years of cigarette smoking, baseline BMI, and prevalent diabetes. Higher intakes of saturated and monounsaturated fats were associated with higher adjusted mean levels of HDL-C, lower mean levels of TGs, and a lower TG:HDL ratio in both women and men. Sex-specific differences were noted for the associations of polyunsaturated fats with lipid levels. In women, higher intakes of both omega-3s and omega-6s PUFAs were associated with lower TG:HDL ratio due mainly to higher HDL levels, while in men, only omega-3s led to a lower TG:HDL ratio. There was no indication that dietary fat of any type was associated with higher mean LDL-C levels.

**FIGURE 5:**
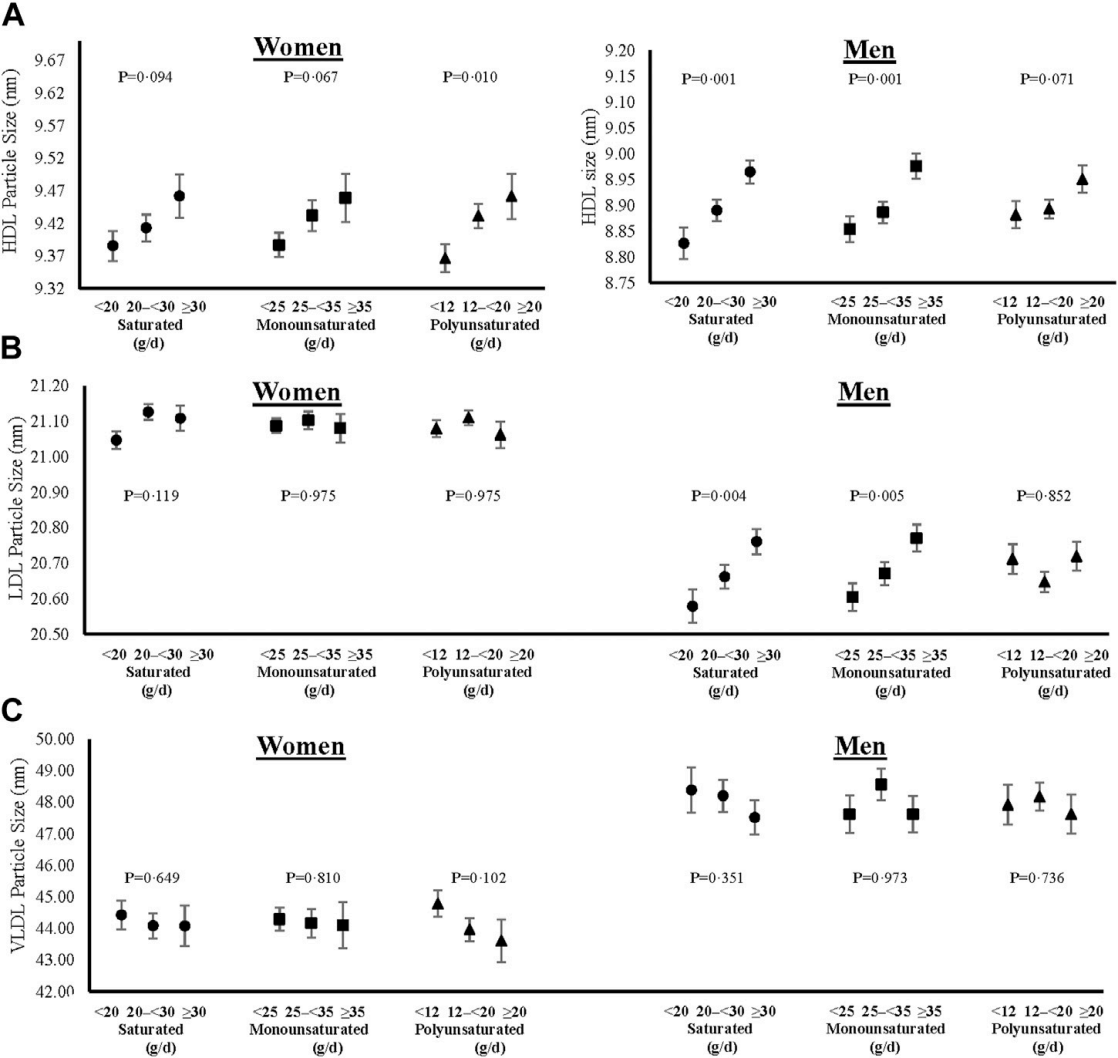
Cross-sectional associations of dietary fat intakes on lipid particle sizes of HDL **(A)**, LDL **(B)** and VLDL **(C)** in women and men. All models were adjusted for age, weight-adjusted carbohydrate intakes, HEI 2015 scores, use of lipid-lowering medications, pack years of cigarette smoking, baseline BMI, and prevalent diabetes. HDL = high density lipoprotein, LDL = low density lipoprotein, VLDL = very low-density lipoprotein, HEI = Healthy Eating Index, and BMI = body mass index.

**FIGURE 6:**
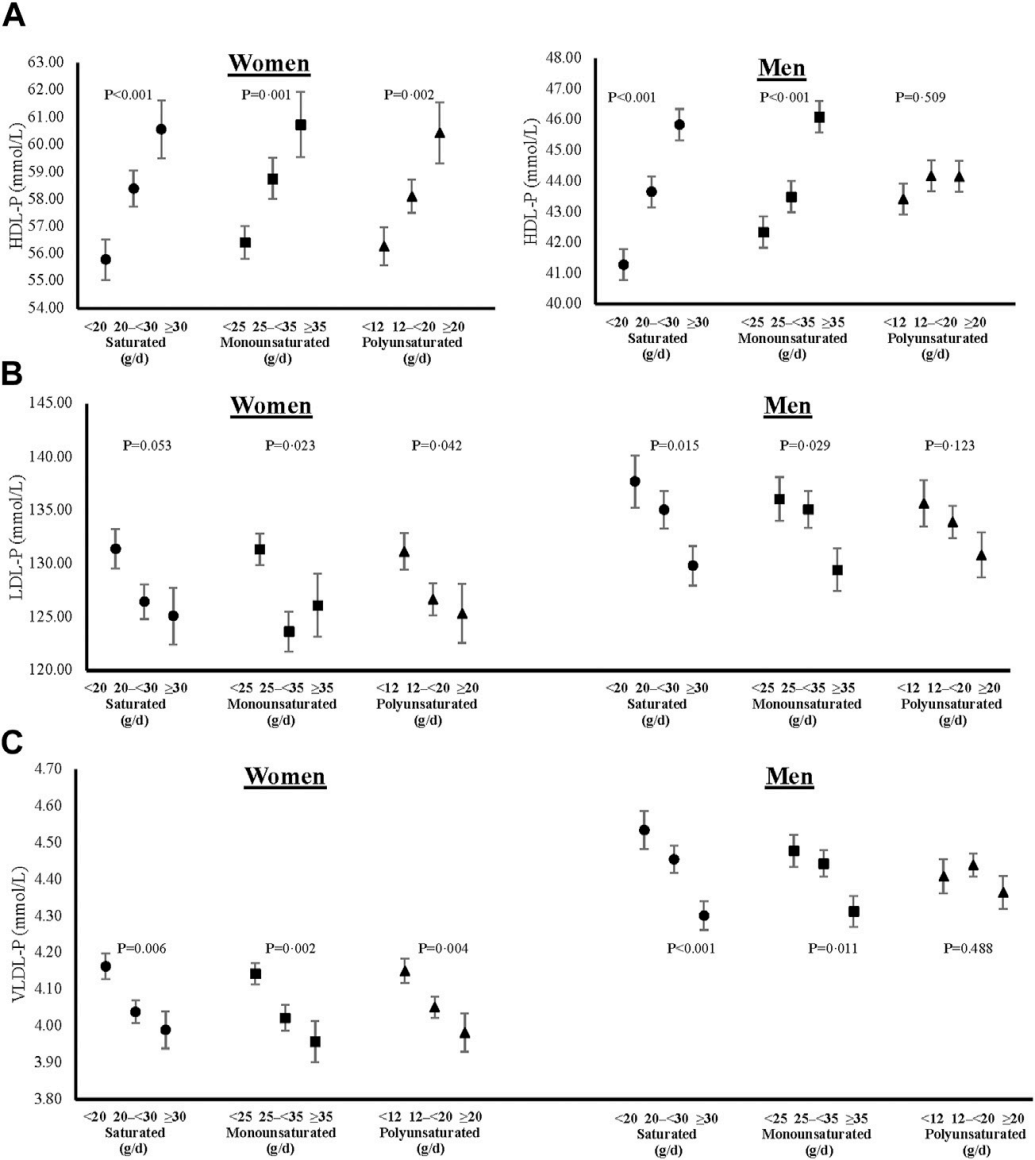
Cross-sectional associations of dietary fat intakes on lipid particle concentrations of HDL **(A)**, LDL **(B)** and VLDL **(C)** in women and men. All models were adjusted for age, weight-adjusted carbohydrate intakes, HEI 2015 scores, use of lipid-lowering medications, pack years of cigarette smoking, baseline BMI, and prevalent diabetes. HDL-P = high density lipoprotein particle concentration, LDL-P = low density lipoprotein particle concentration, VLDL-P = very low-density lipoprotein particle concentration, HEI = Healthy Eating Index, and BMI = body mass index.

In addition to lipid levels, we also show cross-sectional sex-specific associations between dietary fats and adjusted mean lipoprotein particle sizes (Figure 5) and concentrations (Figure 6). Overall, higher intakes of all types of dietary fat tended to be positively associated with HDL particle size in both women and men. However, higher intakes of saturated and monounsaturated fats were associated with beneficial higher mean LDL particle sizes in men only. Regarding lipoprotein concentrations, higher intakes of saturated and monounsaturated fat intakes were favorably associated with all lipoprotein concentrations (HDL, LDL, and VLDL) in both women and men. Women with higher intakes of polyunsaturated fats also had higher mean HDL and lower mean LDL and VLDL particle concentrations, while men had no different concentration levels between the intake categories of polyunsaturated fats.

### Dietary fat intakes and adiposity

After adjusting for confounding by age, carbohydrate intakes, HEI scores, use of lipid-lowering medications, pack-years of cigarette smoking, and prevalent diabetes, these results show that higher (vs. lower) intakes of any type of fat were associated with lower BMI levels, a lower percent body fat, and a smaller waist-to-height ratio in women. Only saturated fat was inversely associated with all three measures of adiposity in men. Finally, monounsaturated fats and omega-6 PUFAs were inversely associated with BMI among men (Table 2).

**TABLE 2:** Sex-specific mean levels of adiposity associated with weight-adjusted intakes of dietary fats.

	Women				Men			
		BMI (kg/m^2^)	% body fat	WHtR		BMI (kg/m^2^)	% body fat	WHtR
	N	Mean (SE)	Mean (SE)	Mean (SE)	N	Mean (SE)	Mean (SE)	Mean (SE)
**Saturated fat** (g/day)								
<20	492	27.7 (0.25)	38.2 (0.34)	0.591 (0.004)	238	28.2 (0.25)	28.7 (0.45)	0.577 (0.004)
20–<30	538	25.9 (0.21)	35.9 (0.29)	0.564 (0.004)	350	27.4 (0.18)	27.8 (0.32)	0.569 (0.003)
≥30	232	26.2 (0.36)	36.2 (0.49)	0.572 (0.006)	372	27.1 (0.20)	27.2 (0.35)	0.563 (0.003)
**P**_**trend**_		<0.001	<0.002	0.002		0.002	0.025	0.010
**Monounsaturated fat** (g/day)								
<25	679	27.1 (0.20)	37.5 (0.27)	0.583 (0.003)	301	28.0 (0.21)	28.3 (0.38)	0.573 (0.003)
25–<35	415	26.0 (0.24)	36.1 (0.34)	0.568 (0.004)	362	27.3 (0.18)	27.7 (0.32)	0.566 (0.003)
≥35	168	26.4 (0.41)	36.3 (0.56)	0.569 (0.007)	297	27.6 (0.21)	27.4 (0.38)	0.567 (0.003)
**P**_**trend**_		0.020	0.010	0.020		0.029	0.130	0.229
**Polyunsaturated fat** (g/day)								
<12	468	27.2 (0.23)	37.5 (0.32)	0.584 (0.004)	246	28.0 (0.23)	28.3 (0.41)	0.573 (0.003)
12–<20	612	26.2 (0.20)	36.4 (0.27)	0.570 (0.003)	468	27.3 (0.16)	27.5 (0.28)	0.567 (0.002)
≥20	182	26.6 (0.38)	36.7 (0.52)	0.575 (0.006)	246	27.4 (0.22)	27.8 (0.40)	0.567 (0.003)
**P**_**trend**_		0.048	0.053	0.056		0.075	0.370	0.226
**Omega-3 PUFAs** (g/day)								
<1.0	295	27.5 (0.29)	38.0 (0.40)	0.590 (0.005)	149	27.8 (0.28)	28.3 (0.51)	0.569 (0.004)
1.0–<2.0	756	26.3 (0.18)	36.4 (0.24)	0.572 (0.003)	568	27.4 (0.14)	27.7 (0.25)	0.569 (0.002)
≥2.0	211	26.5 (0.34)	36.8 (0.47)	0.574 (0.006)	243	27.4 (0.22)	27.8 (0.40)	0.568 (0.003)
**P**_**trend**_		0.015	0.026	0.020		0.372	0.484	0.785
**Omega-6 PUFAs** (g/day)								
<10	394	27.3 (0.25)	37.7 (0.35)	0.586 (0.004)	211	28.0 (0.24)	28.3 (0.44)	0.574 (0.004)
10–<15	514	26.2 (0.21)	36.4 (0.30)	0.571 (0.004)	337	27.4 (0.18)	27.6 (0.33)	0.568 (0.003)
≥15	354	26.5 (0.27)	36.7 (0.37)	0.574 (0.005)	412	27.3 (0.17)	27.7 (0.31)	0.566 (0.003)
**P**_**trend**_		0.028	0.035	0.039		0.025	0.418	0.110

Models were adjusted for age, weight-adjusted carbohydrate intake, HEI, 2015 scores, use of lipid-lowering medications, pack-years of cigarette smoking, and prevalent diabetes. BMI, body mass index; WHtR, waist: height ratio; PUFA, polyunsaturated fatty acids; and HEI, healthy eating index.

### Dietary fat intake, inflammatory markers, and fasting glucose

There were no associations between saturated or monounsaturated fats and inflammatory biomarkers or fasting glucose among women. However, among men, there was an inverse association between saturated fat consumption and log-transformed fibrinogen levels; among women, omega-3 PUFAs were inversely associated with IL-6. There were no associations between any dietary fats and fasting glucose levels (Table 3).

**TABLE 3:** Sex-specific mean levels of inflammatory biomarkers and fasting glucose levels associated with weight-adjusted intakes of dietary fats.

	Women						Men					
	Inflammation			Glucose			Inflammation			Glucose		
	Log (Interleukin-6) (pg/mL)		Log (Fibrinogen) (mg/100 mL)	Fasting glucose (mg/dL)			Log (Interleukin-6) (pg/mL)		Log (Fibrinogen) (mg/100 mL)	Fasting glucose (mg/dL)		
	N	Mean (SE)	Mean (SE)	N		Mean (SE)	N	Mean (SE)	Mean (SE)	N		Mean (SE)
**Saturated fat (**g/day)												
<20	418	1.30 (0.02)	5.81 (0.01)		451	93.6 (0.46)	222	1.35 (0.03)	5.79 (0.01)		225	99.9 (0.68)
20–<30	468	1.30 (0.02)	5.79 (0.01)		521	93.9 (0.38)	335	1.36 (0.02)	5.79 (0.01)		348	99.7 (0.47)
≥30	204	1.30 (0.03)	5.79 (0.01)		226	94.7 (0.64)	374	1.34 (0.02)	5.75 (0.01)		377	99.6 (0.51)
**P**_**trend**_		0.975	0.317			0.252		0.777	0.023			0.750
**Monounsaturated fat** (g/day)												
<25	578	1.32 (0.02)	5.81 (0.01)		633	93.6 (0.36)	287	1.37 (0.03)	5.78 (0.01)		290	99.1 (0.56)
25–<35	362	1.26 (0.02)	5.79 (0.01)		398	94.2 (0.44)	350	1.34 (0.02)	5.78 (0.01)		362	100.3 (0.46)
≥35	150	1.31 (0.04)	5.79 (0.02)		167	94.6 (0.72)	294	1.34 (0.02)	5.76 (0.01)		298	99.6 (0.55)
**P**_**trend**_		0.284	0.219			0.185		0.502	0.091			0.631
**Polyunsaturated fat (**g/day)												
<12	395	1.35 (0.02)	5.80 (0.01)		434	93.8 (0.43)	232	1.35 (0.03)	5.77 (0.01)		231	100.4 (0.60)
12–<20	535	1.26 (0.02)	5.80 (0.01)		586	94.1 (0.36)	462	1.35 (0.02)	5.78 (0.01)		475	99.1 (0.40)
≥20	160	1.30 (0.03)	5.79 (0.01)		178	93.8 (0.67)	237	1.34 (0.03)	5.77 (0.01)		244	100.3 (0.58)
**P**_**trend**_		0.058	0.896			0.854		0.819	0.789			0.971
**Omega-3 PUFAs** (g/day)												
<1.0	243	1.34 (0.03)	5.80 (0.01)		264	93.3 (0.55)	136	1.37 (0.03)	5.76 (0.01)		137	99.7 (0.76)
1.0–<2.0	662	1.30 (0.02)	5.80 (0.01)		727	94.4 (0.32)	554	1.35 (0.02)	5.78 (0.01)		577	99.4 (0.36)
≥2.0	185	1.25 (0.03)	5.79 (0.01)		207	93.4 (0.61)	241	1.33 (0.03)	5.78 (0.01)		236	100.5 (0.58)
**P**_**trend**_		0.035	0.660			0.837		0.370	0.344			0.301
**Omega-6 PUFAs (**g/day)												
<10	331	1.36 (0.02)	5.80 (0.01)		369	93.6 (0.46)	196	1.35 (0.03)	5.77 (0.01)		192	100.3 (0.66)
10–<15	452	1.26 (0.02)	5.80 (0.01)		487	94.2 (0.39)	338	1.34 (0.02)	5.78 (0.01)		352	98.6 (0.47)
≥15	307	1.30 (0.02)	5.79 (0.01)		342	94.0 (0.48)	397	1.36 (0.02)	5.77 (0.01)		406	100.4 (0.45)
**P**_**trend**_		0.061	0.326			0.516		0.736	0.695			0.458

Models were adjusted for age, weight-adjusted carbohydrate intake, HEI, 2015 scores, use of lipid-lowering medications, pack-years of cigarette smoking, and prevalent diabetes. Inflammatory marker models were additionally adjusted for baseline BMI. Log, logarithmic transformed; PUFA, polyunsaturated fat; HEI, healthy eating index; and BMI, body mass index.

## Discussion

In this US community-based cohort of mainly Caucasian women and men, we found no evidence to support an adverse relationship between any type of dietary fat and several cardiometabolic risk factors, including lipids, adiposity, inflammation, and glucose. We found that saturated and monounsaturated fat intakes tended to be favorably associated with TG:HDL ratio in both women and men. However, sex-specific differences were noted for the associations of polyunsaturated fat intake on lipid levels. Women who consumed more omega-3 and omega-6 PUFAs had a lower mean TG:HDL ratio, while among men, only omega-3 PUFAs were inversely associated.

In addition to these associations with serum lipid levels, we also found that all types of dietary fat were associated with larger protective HDL particle sizes in both men and women. Women with higher intakes of all fat types also had higher concentrations of HDL particles, while for men, this was the case only in association with saturated and monounsaturated fats. Further, both saturated and monounsaturated fat consumption among men was associated with larger LDL particle sizes which are less atherogenic than the small dense particles that are more prone to oxidation (DiNicolantonio and O’Keefe, 2018; Forouhi et al., 2018). In general, dietary fats tended to be associated with lower LDL and VLDL particle concentrations. Once again, this was the case for all types of dietary fats among women, while in men, these findings were evident only in associations with saturated and monounsaturated fats. We also noted that a higher intake of saturated fat was associated with less adiposity in both women and men. In addition, higher saturated fat intakes were associated with lower abdominal adiposity in both sexes, while unsaturated fat was only associated with less adiposity in women. Lastly, there were a few differences in the associations between dietary fats and inflammatory markers between women and men. In women, there was some evidence of a beneficial association between polyunsaturated fat and IL-6 levels, whereas saturated fat was inversely associated with fibrinogen levels in men.

Our results contradict the long-held belief that high saturated fat is associated with an atherogenic lipid profile. The rationale for this was based on selected trials (Hooper et al., 2020), despite recent clinical and epidemiological evidence showing otherwise. The LIPGENE study, which is the largest diet intervention study among weight-stable individuals with metabolic syndrome from 18 European countries, also failed to show adverse associations with several lipid and apolipoprotein concentrations after reducing saturated fat intakes. Authors suggested that the absence of a reduction in LDL was due to individuals with higher BMI exhibiting smaller than expected reductions in LDL-C in response to reductions in dietary saturated fat intakes (Tierney et al., 2011). However, a subsequent randomized control trial of overweight and obese individuals without diabetes in the absence of weight loss showed that a high *versus* low saturated fat diet (mainly from dairy sources) had no differences in LDL, HDL, or TGs levels after adjustment for BMI (Chiu et al., 2014). Consistent with our results, the LIPGENE study also showed that high saturated or monounsaturated fat diets were associated with a lower atherogenic index (Tierney et al., 2011). Previous analyses from the PURE study with >100,000 participants showed that diets rich in saturated fat were associated with higher LDL levels but also with higher levels of HDL, lower TGs, and a lower apolipoprotein B: apolipoprotein A ratio (Mente et al., 2017). Lastly, clinical trials have shown that monounsaturated fat-rich meals could form larger chylomicrons, thus increasing the clearance of TGs (DiNicolantonio and O’Keefe, 2018).

In the present study, sex was an important determinant for the associations between polyunsaturated fats and lipid profiles. In women, omega-3 and omega-6 PUFAs were associated with higher mean levels of HDL and lower mean levels of TG:HDL ratio, while in men, omega-3s only led to a lower TG:HDL ratio because of concurrent higher HDL and lower TGs means. Overall, information on the sex-specific differences in the associations of various types of fats on lipids is limited. Consistent with our results, in the LIPGENE study, omega-3 PUFA supplementation led to reduced TGs in men only. Potential mechanisms could be the longer residence time of VLDL TGs in men enabling greater clearance by omega-3 PUFAs and greater metabolic utilization of long-chain omega-3s in men (Tierney et al., 2011). Further, it is known that men tend to have higher concentrations of small dense LDL particles, higher LDL levels, and TG levels, all features of an atherogenic lipoprotein phenotype compared with that observed in premenopausal women (Swiger et al., 2014). A clinical trial showed that after excluding individuals with atherogenic lipoprotein profiles, increasing the dietary intake of omega-3s from foods led to a smaller proportion of small dense LDL particles and lower concentrations of TGs, particularly in men, after adjusting for baseline values. The proportion of HDL_2_ was also increased after increasing omega-3s in both women and men (Griffin et al., 2006), which is consistent with the present findings.

Dietary fats have been associated with consuming energy-dense foods and, as a result, are hypothesized to increase body fat. However, this hypothesis fails to consider the importance of mechanisms involved in fuel partitioning (Manco et al., 2004) and satiety signals, and the divergent effects of different types of fat on lipid metabolism (Forouhi et al., 2018) and the gut microbiome (Machate et al., 2020). Our study differs from those of earlier studies. For example, a previous review suggested that the anti-obesity effects of omega-3s PUFAs were limited to men (Buckley and Howe, 2009). However, in these current analyses, the associations between omega-3 PUFAs and body fat were stronger among women than among men. Finally, our findings on the associations of unsaturated fats on measures of body fat support previous evidence showing that monounsaturated and polyunsaturated fat-enriched diets or meals may increase fat oxidation and energy expenditure while suppressing appetite and visceral fat deposition and beneficially modulate gut microbiota (Buckley and Howe, 2009; Gillingham et al., 2011; Beulen et al., 2018; Cândido et al., 2018; Tutunchi et al., 2020). Lastly, in the LIPGENE study, leptin, a major appetite hormone, was not affected by diets high in any type of fat (Tierney et al., 2011).

In the current study, saturated and monounsaturated fats were inversely associated with fibrinogen levels in men, while omega-3 and omega-6s were inversely associated with IL-6 in women. Previous research has suggested that meals rich in dietary fats may stimulate the innate immune response, thereby promoting inflammation (Fritsche, 2015). In a randomized cross-over study, investigators failed to find any effect of saturated or other fats on circulating inflammatory biomarkers (Voon et al., 2011). The variable associations of dietary fats on inflammation across studies could also be due to dissimilar effects of different dietary fats and their food sources, which could impact pathways such as the gut microbiome differently (Fritsche, 2015). A recent publication from the Framingham Study showed that higher intakes of saturated fats from dairy-derived sources, but not from other sources, were strongly inversely associated with biomarkers of inflammation (Yuan et al., 2022). Lastly, concerns have been raised that an excess of linoleic acid, an essential omega-6 polyunsaturated fat, might promote inflammation and LDL oxidation; however, there is still no consensus on these effects (Innes and Calder, 2018). The American Heart Association concluded that there is little direct evidence to support the pro-inflammatory role of linoleic acid (Harris et al., 2009).

Strengths of the present study include the longer-term dietary intakes that were determined by averaging 3-day food diaries over 8 years. Although dietary records are generally considered a more accurate means of assessing individual dietary intakes (Hunter et al., 1992; Høidrup et al., 2002), we cannot rule out the recall bias from self-reported data. Despite adjusting for several carefully collected risk factors, we cannot rule out residual confounding, particularly with respect to inter-correlated dietary factors. In this study, monounsaturated fat intake was highly correlated with saturated fat (Pearson r = 0.87), which likely limits our ability to assess the independent contribution of these two types of fats. Another limitation of this study is the cross-sectional analysis of lipid particles; therefore, future prospective studies are needed to confirm these results. Lastly, the Framingham Offspring Cohort consists of mainly Caucasian participants, thus limiting the generalizability of these results to a multi-ethnic population.

In summary, we found no evidence to support an adverse relationship between dietary fats and several surrogate markers of cardiometabolic health. Importantly, these findings show that higher intakes of saturated and monounsaturated fat were associated with a less atherogenic lipid profile. Further, saturated fat intakes were associated with lower levels of body fat and a lower waist-to-height ratio in both men and women. There were only limited associations of dietary fats on inflammatory biomarkers and none on fasting glucose. Finally, there were no adverse associations of either monounsaturated or polyunsaturated fats on any of these cardiometabolic outcomes.

## Data Availability

The de-identified Framingham datasets analyzed for this study can be found through the BIOLINCC repository: https://biolincc.nhlbi.nih.gov/studies/framoffspring/. Use of other data requires submission and approval of a research proposal to the Framingham executive committee.

## Abbreviations

BMI, body mass index; CRP, c-reactive protein; CVD, cardiovascular disease; Eq, equivalents; Ex, exam; HDL, high density lipoprotein; HEI, Healthy Eating Index; IL-6, interleukin-6; LDL, low density lipoprotein; Log, logarithmic transformed; MET, metabolic; Oz, ounces; PUFA, polyunsaturated fatty acids; T2DM, type 2 diabetes mellitus; TG, triglycerides; TG:HDL, triglyceride to high density lipoprotein ratio; VLDL, very low-density lipoprotein; WHtR, waist-to-height ratio.

## References

[B1] Beulen Y. Martínez-González M. A. Van de Rest O. Salas-Salvadó J. Sorlí J. V. Gómez-Gracia E. (2018). Quality of dietary fat intake and body weight and obesity in a mediterranean population: Secondary analyses within the PREDIMED trial. Nutrients 10, 2011. 10.3390/nu10122011 30572588 PMC6315420

[B2] Buckley J. D. Howe P. R. C. (2009). Anti-obesity effects of long-chain omega-3 polyunsaturated fatty acids. Obes. Rev. 10, 648–659. 10.1111/j.1467-789X.2009.00584.x 19460115

[B3] Cândido F. G. Valente F. X. Grześkowiak Ł. M. Moreira A. P. B. Rocha D. M. U. P. Alfenas R. de C. G. (2018). Impact of dietary fat on gut microbiota and low-grade systemic inflammation: Mechanisms and clinical implications on obesity. Int. J. Food Sci. Nutr. 69, 125–143. 10.1080/09637486.2017.1343286 28675945

[B4] Chen Y. Chang Z. Liu Y. Zhao Y. Fu J. Zhang Y. (2022). Triglyceride to high-density lipoprotein cholesterol ratio and cardiovascular events in the general population: A systematic review and meta-analysis of cohort studies. Nutr. Metabolism Cardiovasc. Dis. 32, 318–329. 10.1016/j.numecd.2021.11.005 34953633

[B5] Chiu S. Williams P. T. Dawson T. Bergman R. N. Stefanovski D. Watkins S. M. (2014). Diets high in protein or saturated fat do not affect insulin sensitivity or plasma concentrations of lipids and lipoproteins in overweight and obese adults. J. Nutr. 144, 1753–1759. 10.3945/jn.114.197624 25332473 PMC4195419

[B6] DiNicolantonio J. J. O’Keefe J. H. (2018). Effects of dietary fats on blood lipids: A review of direct comparison trials. Open Heart 5, e000871. 10.1136/openhrt-2018-000871 30094038 PMC6074619

[B7] Forouhi N. G. Krauss R. M. Taubes G. Willett W. (2018). Dietary fat and cardiometabolic health: Evidence, controversies, and consensus for guidance. BMJ 361, k2139. 10.1136/bmj.k2139 29898882 PMC6053258

[B8] Fritsche K. L. (2015). The science of fatty acids and inflammation. Adv. Nutr. 6, 293S–301S. 10.3945/an.114.006940 25979502 PMC4424767

[B9] Gerrior S. Juan W. Basiotis P. (2006). An easy approach to calculating estimated energy requirements. Prev. Chronic Dis. 3, A129. 16978504 PMC1784117

[B10] Gillingham L. G. Harris-Janz S. Jones P. J. H. (2011). Dietary monounsaturated fatty acids are protective against metabolic syndrome and cardiovascular disease risk factors. Lipids 46, 209–228. 10.1007/s11745-010-3524-y 21308420

[B11] Griffin M. D. Sanders T. A. Davies I. G. Morgan L. M. Millward D. J. Lewis F. (2006). Effects of altering the ratio of dietary n−6 to n−3 fatty acids on insulin sensitivity, lipoprotein size, and postprandial lipemia in men and postmenopausal women aged 45–70 y: the OPTILIP Study. Am. J. Clin. Nutr. 84, 1290–1298. 10.1093/ajcn/84.6.1290 17158408

[B12] Harris W. S. Mozaffarian D. Rimm E. Kris-Etherton P. Rudel L. L. Appel L. J. (2009). Omega-6 fatty acids and risk for cardiovascular disease: A science advisory from the American heart association nutrition subcommittee of the Council on nutrition, physical activity, and metabolism; Council on cardiovascular nursing; and Council on epidemiology and prevention. Circulation 119, 902–907. 10.1161/CIRCULATIONAHA.108.191627 19171857

[B13] Høidrup S. Andreasen A. H. Osler M. Pedersen A. N. Jørgensen L. M. Jørgensen T. (2002). Assessment of habitual energy and macronutrient intake in adults: Comparison of a seven day food record with a dietary history interview. Eur. J. Clin. Nutr. 56, 105–113. 10.1038/sj.ejcn.1601292 11857043

[B14] Hooper L. Martin N. Jimoh O. F. Kirk C. Foster E. Abdelhamid A. S. (2020). Reduction in saturated fat intake for cardiovascular disease. Cochrane Database Syst. Rev. 5, CD011737. 10.1002/14651858.cd011737.pub3 32428300 PMC7388853

[B15] Hunter D. J. Rimm E. B. Sacks F. M. Stampfer M. J. Colditz G. A. Litin L. B. (1992). Comparison of measures of fatty acid intake by subcutaneous fat aspirate, food frequency questionnaire, and diet records in a free-living population of US men. Am. J. Epidemiol. 135, 418–427. 10.1093/oxfordjournals.aje.a116302 1550093

[B16] Innes J. K. Calder P. C. (2018). Omega-6 fatty acids and inflammation. Prostagl. Leukot. Essent. Fat. Acids 132, 41–48. 10.1016/j.plefa.2018.03.004 29610056

[B17] Kannel W. B. Belanger A. D’Agostino R. Israel I. (1986). Physical activity and physical demand on the job and risk of cardiovascular disease and death: The Framingham Study. Am. Heart J. 112, 820–825. 10.1016/0002-8703(86)90480-1 3766383

[B18] Kannel W. B. Sorlie P. (1979). Some health benefits of physical activity: The Framingham Study. Arch. Intern Med. 139, 857–861. 10.1001/archinte.1979.03630450011006 464698

[B19] Knopp R. H. Paramsothy P. Retzlaff B. M. Fish B. Walden C. Dowdy A. (2005). Gender differences in lipoprotein metabolism and dietary response: Basis in hormonal differences and implications for cardiovascular disease. Curr. Atheroscler. Rep. 7, 472–479. 10.1007/s11883-005-0065-6 16256006

[B20] Krauss R. M. (2022). Small dense low-density lipoprotein particles: Clinically relevant? Curr. Opin. Lipidol. 33, 160–166. 10.1097/MOL.0000000000000824 35276699 PMC9197986

[B21] Krebs-Smith S. M. Pannucci T. E. Subar A. F. Kirkpatrick S. I. Lerman J. L. Tooze J. A. (2018). Update of the healthy eating index: HEI-2015. J. Acad. Nutr. Diet. 118, 1591–1602. 10.1016/j.jand.2018.05.021 30146071 PMC6719291

[B22] Lukaski H. C. Bolonchuk W. W. Hall C. B. Siders W. A. (1986). Validation of tetrapolar bioelectrical impedance method to assess human body composition. J. Appl. Physiology 60, 1327–1332. 10.1152/jappl.1986.60.4.1327 3700310

[B23] Machate D. J. Figueiredo P. S. Marcelino G. Guimarães R. C. A. Hiane P. A. Bogo D. (2020). Fatty acid diets: Regulation of gut microbiota composition and obesity and its related metabolic dysbiosis. Int. J. Mol. Sci. 21, 4093. 10.3390/ijms21114093 32521778 PMC7312778

[B24] Manco M. Calvani M. Mingrone G. (2004). Effects of dietary fatty acids on insulin sensitivity and secretion. Diabetes, Obes. Metabolism 6, 402–413. 10.1111/j.1462-8902.2004.00356.x 15479216

[B25] McNamara J. R. Schaefer E. J. (1987). Automated enzymatic standardized lipid analyses for plasma and lipoprotein fractions. Clin. Chim. Acta 166, 1–8. 10.1016/0009-8981(87)90188-4 3608193

[B26] Mensink R. P. Zock P. L. Kester A. D. Katan M. B. (2003). Effects of dietary fatty acids and carbohydrates on the ratio of serum total to HDL cholesterol and on serum lipids and apolipoproteins: A meta-analysis of 60 controlled trials. Am. J. Clin. Nutr. 77, 1146–1155. 10.1093/ajcn/77.5.1146 12716665

[B27] Mente A. Dehghan M. Rangarajan S. McQueen M. Dagenais G. Wielgosz A. (2017). Association of dietary nutrients with blood lipids and blood pressure in 18 countries: A cross-sectional analysis from the PURE study. Lancet Diabetes & Endocrinol. 5, 774–787. 10.1016/S2213-8587(17)30283-8 28864143

[B28] Parhofer K. G. (2015). Interaction between glucose and lipid metabolism: More than diabetic dyslipidemia. Diabetes Metab. J. 39, 353–362. 10.4093/dmj.2015.39.5.353 26566492 PMC4641964

[B29] Schakel S. F. Sievert Y. A. Buzzard I. M. (1988). Sources of data for developing and maintaining a nutrient database. J. Am. Dietetic Assoc. 88, 1268–1271. 10.1016/s0002-8223(21)07997-9 3171020

[B30] Swiger K. J. Martin S. S. Blaha M. J. Toth P. P. Nasir K. Michos E. D. (2014). Narrowing sex differences in lipoprotein cholesterol subclasses following mid‐life: The very large database of lipids (VLDL-10B). J. Am. Heart Assoc. 3, e000851. 10.1161/JAHA.114.000851 24755154 PMC4187479

[B31] Tierney A. C. McMonagle J. Shaw D. I. Gulseth H. L. Helal O. Saris W. H. M. (2011). Effects of dietary fat modification on insulin sensitivity and on other risk factors of the metabolic syndrome-- LIPGENE: A European randomized dietary intervention study. Int. J. Obes. 35, 800–809. 10.1038/ijo.2010.209 20938439

[B32] Tutunchi H. Ostadrahimi A. Saghafi-Asl M. (2020). The effects of diets enriched in monounsaturated oleic acid on the management and prevention of obesity: A systematic review of human intervention studies. Adv. Nutr. 11, 864–877. 10.1093/advances/nmaa013 32135008 PMC7360458

[B33] U.S. Department of AgricultureU.S. Department of Health and Human Services (2020). Dietary guidelines for Americans. 9th Edition. Available at: https://www.dietaryguidelines.gov/ (Accessed April 4, 2023).

[B34] Varlamov O. Bethea C. L. Roberts C. T. (2015). Sex-specific differences in lipid and glucose metabolism. Front. Endocrinol. 5, 241. 10.3389/fendo.2014.00241 PMC4298229 25646091

[B35] Voon P. T. Ng T. K. W. Lee V. K. M. Nesaretnam K. (2011). Diets high in palmitic acid (16:0), lauric and myristic acids (12:0 + 14:0), or oleic acid (18:1) do not alter postprandial or fasting plasma homocysteine and inflammatory markers in healthy Malaysian adults. Am. J. Clin. Nutr. 94, 1451–1457. 10.3945/ajcn.111.020107 22030224

[B36] Yiannakou I. Yuan M. Zhou X. Singer M. R. Moore L. L. (2022). Saturated and unsaturated dietary fats and cardiometabolic risk in the Framingham Offspring Study. SSRN J. 10.2139/ssrn.4198093

[B37] Yuan M. Singer M. R. Pickering R. T. Moore L. L. (2022). Saturated fat from dairy sources is associated with lower cardiometabolic risk in the Framingham Offspring Study. Am. J. Clin. Nutr. 116, 1682–1692. 10.1093/ajcn/nqac224 36307959 PMC9761752

