# Supplementary material for: Dietary fat intakes, lipid profiles, adiposity, inflammation, and glucose in women and men in the Framingham Offspring Cohort
Source: Front Physiol. 2023 May 3;14:1144200. doi: 10.3389/fphys.2023.1144200 (PMC10206522; doi:10.3389/fphys.2023.1144200)
Supplement: Supplementary file 1 [file DataSheet1.docx]

Supplementary Material

**Dietary fat intakes, adiposity, inflammation, glucose and lipid profiles in women and men in Framingham Offspring Cohort.**

**Ioanna Yiannakou, Mengjie Yuan, Xinyi Zhou, Martha R. Singer, Lynn L. Moore***

***Correspondence:** llmoore@bu.edu

[Supplementary Table 1 2](#_Toc124432069)

[Supplementary Table 2 3](#_Toc124432070)

| Supplementary Table 1 **Sex-specific characteristics of participants according to weight-adjusted intakes of monounsaturated fats** | | | | | | | | |
| --- | --- | --- | --- | --- | --- | --- | --- | --- |
|  | **Women** | | |  | **Men** | | | |
|  | **Monounsaturated fat intake (g/day)** | | |  | **Monounsaturated fat intake (g/day)** | | | |
|  | **<25** (n=704) | **25-<35** (n=424) | **≥35** (n=172) |  | **<25** (n=347) | **25-<35** (n=410) | **≥35** (n=334) | |
| **Participant Characteristics** | Mean (SE) | Mean (SE) | Mean (SE) |  | Mean (SE) | Mean (SE) | Mean (SE) | |
| Age | 56.6 (0.4) | 54.9 (0.5) | 52.1 (0.7) |  | 59.2 (0.5) | 55.6 (0.5) | 53.8 (0.5) | |
| BMI (kg/m^2^) | 27.2 (0.2) | 25.3 (0.2) | 25.2 (0.4) |  | 29.0 (0.2) | 27.6 (0.2) | 27.2 (0.2) | |
| Smoking (packyears) | 21.8 (1.0) | 22.0 (1.3) | 23.4 (1.9) |  | 27.7 (1.4) | 28.4 (1.3) | 31.8 (1.5) | |
| Physical activity index (MET-eq/day) | 14.6 (0.3) | 14.0 (0.4) | 13.6 (0.6) |  | 14.8 (0.5) | 15.0 (0.4) | 15.7 (0.5) | |
| Alcohol, g/day (current drinkers) | 9.1 (0.5) | 9.7 (0.6) | 10.5 (0.9) |  | 15.7 (1.1) | 19.0 (1.0) | 18.8 (1.1) | |
| HEI-2015 score | 60.2 (0.4) | 56.1 (0.5) | 54.7 (0.9) |  | 58.3 (0.6) | 54.7 (0.5) | 51.7 (0.6) | |
| Total fat (g/day, weight-adjusted) | 73.5 (1.0) | 75.6 (1.2) | 74.7 (1.9) |  | 74.3 (1.4) | 73.8 (1.3) | | 76.0 (1.4) |
| Saturated | 17.8 (0.20) | 26.9 (0.25) | 36.0 (0.40) |  | 17.8 (0.36) | 27.6 (0.32) | | 38.9 (0.36) |
| Monounsaturated | 19.1 (0.14) | 29.2 (0.18) | 40.7 (0.29) |  | 19.3 (0.25) | 29.9 (0.23) | | 43.2 (0.26) |
| Polyunsaturated fats | 11.4 (0.16) | 16.2 (0.20) | 21.7 (0.32) |  | 11.6 (0.28) | 16.0 (0.25) | | 22.2 (0.28) |
| Omega-3 | 1.2 (0.02) | 1.6 (0.02) | 2.1 (0.04) |  | 1.3 (0.03) | 1.6 (0.03) | | 2.1 (0.03) |
| Omega-6 | 10.1 (0.14) | 14.5 (0.18) | 19.5 (0.29) |  | 10.2 (0.26) | 14.3 (0.23) | | 20.0 (0.26) |
| Food intakes |  |  |  |  |  |  |  | |
| Fruit/Vegetables (cup-eq/day) | 2.9 (0.05) | 3.0 (0.06) | 3.3 (0.10) |  | 3.3 (0.09) | 3.2 (0.08) | 3.4 (0.09) | |
| Meat, poultry, fish (oz-eq/day) | 4.1 (0.07) | 4.5 (0.08) | 5.5 (0.13) |  | 5.3 (0.11) | 5.8 (0.10) | 6.7 (0.12) | |
| Red meats (oz-eq/day) | 1.4 (0.05) | 2.0 (0.06) | 2.7 (0.09) |  | 2.1 (0.09) | 2.9 (0.08) | 3.7 (0.09) | |
| Poultry (oz-eq/day) | 1.5 (0.05) | 1.4 (0.06) | 1.4 (0.09) |  | 1.8 (0.08) | 1.6 (0.07) | 1.7 (0.08) | |
| Fish (oz-eq/day) | 1.1 (0.04) | 1.1 (0.05) | 1.4 (0.09) |  | 1.4 (0.07) | 1.3 (0.07) | 1.3 (0.08) | |
| High omega-3 fish (oz-eq/day) | 0.2 (0.02) | 0.2 (0.02) | 0.2 (0.03) |  | 0.2 (0.03) | 0.3 (0.03) | 0.3 (0.03) | |
| Nuts & seeds (oz-eq/day) | 0.2 (0.02) | 0.5 (0.03) | 0.9 (0.05) |  | 0.3 (0.05) | 0.5 (0.04) | 0.9 (0.05) | |
| Dairy (cup-eq/day) | 1.1 (0.03) | 1.3 (0.04) | 1.4 (0.06) |  | 1.2 (0.05) | 1.5 (0.05) | 1.8 (0.05) | |
| Education level (column %, >high school) | 56.3 | 64.9 | 65.7 |  | 63.1 | 68.1 | 64.7 | |
| Current smokers (column %) | 17.0 | 14.9 | 18.0 |  | 11.2 | 14.6 | 17.1 | |
| Mean fat intakes (exams 3 and 5) were adjusted for weight using residuals from a linear regression model. Analyses were adjusted for age. BMI= body mass index, Mets=metabolic, eq=equivalents, HEI=Healthy Eating Index, and oz=ounces. | | | | | | | | |

| Supplementary Table 2 **Sex-specific characteristics of participants according to weight-adjusted intakes of polyunsaturated fats** | | | | | | | |
| --- | --- | --- | --- | --- | --- | --- | --- |
|  | **Women** | | |  | **Men** | | |
|  | **Polyunsaturated fat intake (g/day)** | | |  | **Polyunsaturated fat intake (g/day)** | | |
|  | **<12** (n=489) | **12-<20** (n=626) | **≥20** (n=185) |  | **<12** (n=281) | **12-<20** (n=537) | **≥20** (n=273) |
| **Participant Characteristics** | Mean (SE) | Mean (SE) | Mean (SE) |  | Mean (SE) | Mean (SE) | Mean (SE) |
| Age | 56.7 (0.4) | 55.2 (0.4) | 53.0 (0.7) |  | 58.5 (0.6) | 56.1 (0.4) | 54.0 (0.6) |
| BMI (kg/m^2^) | 27.5 (0.2) | 25.7 (0.2) | 25.4 (0.4) |  | 28.9 (0.2) | 27.7 (0.2) | 27.2 (0.2) |
| Smoking (packyears) | 23.2 (1.2) | 21.3 (1.1) | 21.9 (1.8) |  | 30.4 (1.5) | 28.8 (1.2) | 28.2 (1.7) |
| Physical activity index (MET-eq/day) | 14.6 (0.3) | 14.3 (0.3) | 13.4 (0.5) |  | 15.7 (0.5) | 14.9 (0.4) | 14.9 (0.5) |
| Alcohol, g/day (current drinkers) | 8.9 (0.6) | 9.8 (0.5) | 10.1 (0.9) |  | 18.9 (1.2) | 17.6 (0.9) | 17.2 (1.2) |
| HEI-2015 score | 57.3 (0.5) | 58.3 (0.5) | 59.9 (0.8) |  | 53.2 (0.6) | 55.0 (0.5) | 56.6 (0.7) |
| Total fat (g/day, weight-adjusted) | 75.1 (1.1) | 73.6 (1.0) | 74.7 (1.9) |  | 73.0 (1.5) | 75.8 (1.1) | 73.9 (1.5) |
| Saturated | 18.9 (0.33) | 24.4 (0.29) | 30.4 (0.54) |  | 21.8 (0.57) | 27.7 (0.41) | 34.6 (0.57) |
| Monounsaturated | 19.1 (0.28) | 26.8 (0.25) | 36.0 (0.46) |  | 22.0 (0.48) | 30.1 (0.35) | 40.5 (0.49) |
| Polyunsaturated fats | 9.3 (0.11) | 15.4 (0.10) | 24.2 (0.18) |  | 9.3 (0.19) | 15.7 (0.13) | 25.5 (0.19) |
| Omega-3 | 1.0 (0.02) | 1.5 (0.02) | 2.3 (0.03) |  | 1.0 (0.03) | 1.6 (0.02) | 2.3 (0.03) |
| Omega-6 | 8.2 (0.10) | 13.8 (0.09) | 21.7 (0.17) |  | 8.2 (0.18) | 14.0 (0.13) | 23.0 (0.18) |
| Food intakes |  |  |  |  |  |  |  |
| Fruit/Vegetables (cup-eq/day) | 2.8 (0.06) | 3.0 (0.05) | 3.5 (0.09) |  | 3.0 (0.09) | 3.3 (0.07) | 3.7 (0.09) |
| Meat, poultry, fish (oz-eq/day) | 4.0 (0.08)) | 4.5 (0.07) | 5.0 (0.13) |  | 5.3 (0.13) | 6.0 (0.09) | 6.5 (0.13) |
| Red meats (oz-eq/day) | 1.7 (0.06) | 1.8 (0.05) | 1.9 (0.10) |  | 2.7 (0.10) | 2.8 (0.07) | 3.1 (0.10) |
| Poultry (oz-eq/day) | 1.3 (0.06) | 1.5 (0.05) | 1.5 (0.09) |  | 1.5 (0.09) | 1.8 (0.06) | 1.8 (0.09) |
| Fish (oz-eq/day) | 1.0 (0.05) | 1.2 (0.04) | 1.5 (0.08) |  | 1.1 (0.08) | 1.4 (0.06) | 1.7 (0.08) |
| High omega-3 fish (oz-eq/day) | 0.2 (0.02) | 0.2 (0.02) | 0.3 (0.03) |  | 0.2 (0.03) | 0.3 (0.02) | 0.3 (0.03) |
| Nuts & seeds (oz-eq/day) | 0.2 (0.03) | 0.4 (0.02) | 0.9 (0.04) |  | 0.3 (0.05) | 0.5 (0.04) | 1.0 (0.05) |
| Dairy (cup-eq/day) | 1.2 (0.04) | 1.2 (0.03) | 1.4 (0.06) |  | 1.4 (0.06) | 1.5 (0.04) | 1.7 (0.06) |
| Education level (column %, >high school) | 53.2 | 61.5 | 75.1 |  | 58.0 | 67.6 | 68.9 |
| Current smokers (column %) | 18.5 | 16.5 | 10.8 |  | 19.2 | 13.6 | 10.6 |
| Mean fat intakes (exams 3 and 5) were adjusted for weight using residuals from a linear regression model. Analyses were adjusted for age. BMI= body mass index, Mets=metabolic, eq=equivalents, HEI=Healthy Eating Index, and oz=ounces. | | | | | | | |
